# Microbiome–Metabolome Crosstalk as a Driver of COVID-19 Severity

**DOI:** 10.3390/medsci14010097

**Published:** 2026-02-17

**Authors:** Patricia Diez-Echave, María Jesús Rodríguez-Sojo, Benita Martin-Castaño, Laura Hidalgo-García, Antonio Jesús Ruiz-Malagon, José Alberto Molina-Tijeras, Anaïs Redruello Romero, Margarita Martínez-Zaldívar, Emilio Mota, Fernando Cobo, Marta Alvarez-Estevez, Federico García, Concepción Morales-García, Silvia Merlos, Paula García-Flores, Manuel Colmenero-Ruiz, María Nuñez, Andrés Ruiz-Sancho, María Elena Rodríguez-Cabezas, Ángel Carazo Gallego, Emilio Fernandez-Varón, José Pérez del Palacio, Javier Martin, Jorge García-García, Rocío Morón, Alba Rodríguez-Nogales, Julio Gálvez

**Affiliations:** 1 Department of Pharmacology, Center for Biomedical Research (CIBM), University of Granada, 18071 Granada, Spain; 2 Instituto de Investigación Biosanitaria de Granada (ibs.GRANADA), 18012 Granada, Spain; 3 Centro de Salud Las Gabias, Distrito Granada-Metropolitano, 18012 Granada, Spain; 4 Centro de Salud “Salvador Caballero”, Distrito Granada-Metropolitano, 18012 Granada, Spain; 5 Servicio Microbiología, Hospital Universitario Virgen de las Nieves, 18014 Granada, Spain; 6 Servicio Microbiología, Hospital Universitario Clínico San Cecilio, 18016 Granada, Spain; 7 CIBER de Enfermedades Infecciosas (CIBER-Infecc), Instituto de Salud Carlos III, 28029 Madrid, Spain; 8 Respiratory Medicine Department, Hospital Universitario Virgen de las Nieves, 18014 Granada, Spain; 9 Servicio de Medicina Intensiva, Hospital Universitario Clínico San Cecilio, 18016 Granada, Spain; 10 Servicio Farmacia Hospitalaria, Hospital Universitario Clínico San Cecilio, 18016 Granada, Spain; 11 Servicio de Infecciosas, Hospital Universitario Clínico San Cecilio, 18016 Granada, Spain; 12 Medical Oncology Unit, University Hospital of Jaén, 23007 Jaén, Spain; 13 Department of Cell Biology and Immunology, Institute of Parasitology and Biomedicine López-Neyra, CSIC, 18016 Granada, Spain; 14 CIBER de Enfermedades Hepáticas y Digestivas (CIBER-EHD), Instituto de Salud Carlos III, 28029 Madrid, Spain

**Keywords:** biomarkers, COVID-19 severity, metabolomic, microbiome, SARS-CoV-2

## Abstract

**Background:** COVID-19, caused by SARS-CoV-2, exhibits highly variable severity, from mild symptoms to respiratory failure and multiorgan dysfunction. Traditional risk factors incompletely explain this heterogeneity, highlighting the potential role of gut microbiota and host metabolomics in modulating immune responses. **Methods:** Thus, this study investigates how gut microbiota variations are associated with plasma metabolite profiles in COVID-19, exploring relationships between microbial and metabolic signatures and disease severity and potential therapeutic targets. In a prospective cohort of 55 patients, stool and plasma samples were analyzed using 16S rRNA sequencing and untargeted LC-HRMS metabolomics. **Results:** Severe COVID-19 was associated with reduced microbial diversity and enrichment of pro-inflammatory taxa, including *Prevotella*, *Alistipes*, *Dialister*, and *Lachnoclostridium*, whereas mild cases showed higher abundance of protective commensals such as *Bacteroides*, *Faecalibacterium*, and *Blautia*. Metabolomic profiling revealed alterations in bile acids, unsaturated fatty acids, tryptophan, and inositol phosphate pathways. Notably, linoleate levels were elevated in severe cases, showing correlations with pro-inflammatory microbes, while acylcarnitines and inositol derivatives were enriched in mild disease. Predictive functional analysis suggested that severe-associated microbes showed enhanced amino acid catabolism, oxidative glucose metabolism, and xenobiotic degradation, which may be linked to host inflammation. **Conclusions:** These findings highlight associations between gut microbiota composition, microbial metabolism, and circulating metabolites in COVID-19 severity. Identified microbial and metabolomic signatures may represent potential candidates to be considered biomarkers and therapeutic targets to modulate disease progression.

## 1. Introduction

Coronavirus disease 2019 (COVID-19), caused by the severe acute respiratory syndrome coronavirus 2 (SARS-CoV-2), has infected over 700 million individuals and led to more than 7 million deaths worldwide as of early 2024 [1]. While most infections are asymptomatic or present with mild-to-moderate respiratory symptoms, a significant proportion of patients develop severe disease marked by pneumonia, acute respiratory distress syndrome (ARDS), and multiorgan failure [2]. Established risk factors—such as advanced age, male sex, obesity, chronic cardiovascular or pulmonary comorbidities, and the presence of autoantibodies against interferons—have been consistently linked to worse outcomes [3,4]. However, these variables alone do not fully explain the striking heterogeneity in disease manifestations and severity observed among patients.

Emerging evidence indicates that COVID-19 severity is largely driven by an imbalanced host immune response, characterized by excessive production of pro-inflammatory cytokines, lymphopenia, depletion of non-classical monocytes, and an increase in immature neutrophils in peripheral blood [5,6]. Additionally, the continuous emergence of viral variants of concern has complicated disease control and challenged the effectiveness of vaccines and antiviral treatments [7]. These findings underscore the need to identify additional host-related and environmental factors that could modulate disease progression and severity. Among these, host metabolomic alterations and gut microbiota composition have emerged as key modulators of immune function and systemic inflammation in COVID-19 [8,9]. Several studies have documented profound gut microbial dysbiosis in severe COVID-19, marked by reduced abundance of beneficial commensals such as *Faecalibacterium prausnitzii* and overgrowth of opportunistic pathogens including *Clostridium hathewayi* and *Coprobacillus* spp., compared to patients with mild disease or uninfected controls [10]. These microbial shifts may amplify systemic inflammation by compromising gut barrier integrity and promoting translocation of pro-inflammatory microbial products, thereby contributing to the cytokine storm and immune dysregulation observed in critical cases [11].

Concurrently, metabolomic analyses have revealed marked disruptions in several metabolic pathways linked to disease severity, including tryptophan metabolism, lipid metabolism, and bile acid transformation [12]. Notably, enhanced activity of the kynurenine pathway—a major route of tryptophan catabolism—has been associated with heightened systemic inflammation and immune dysfunction, correlating positively with disease severity [13]. Alterations in bile acid composition, specifically elevated primary bile acids and reduced secondary bile acids, have also been implicated in perpetuating inflammatory damage [14]. Moreover, recent work has highlighted the role of microbiota-derived metabolites, which differ significantly between COVID-19 patients and healthy controls, suggesting that these compounds may actively influence disease pathogenesis [15].

Despite these advances, most integrative studies to date have focused on cohorts with coexisting conditions, such as Human Immunodeficiency Virus (HIV) infection or Crohn’s disease, where the interplay between microbiota and host metabolism may be confounded by underlying chronic inflammation [16,17]. By contrast, few studies have investigated this microbiome–metabolome axis in patients suffering exclusively from SARS-CoV-2 infection, leaving a critical gap in understanding how gut microbial composition might shape systemic metabolic responses in the absence of other chronic diseases. This distinction is clinically relevant, as it allows for the assessment of virus-induced metabolic and microbial changes without the cofounding effects of pre-existing systemic inflammation, thereby providing a clearer view of SARS-CoV-2-specific effects.

Therefore, the present study aimed not merely to characterize microbiota-derived metabolites but to investigate whether differences in gut microbiota composition could influence the abundance of key plasma metabolites in COVID-19 patients. By integrating taxonomic and metabolomic data, this approach seeks to provide a broader perspective on the microbiome–metabolome–severity axis in COVID-19, offering potential insights into novel biomarkers and therapeutic strategies to mitigate disease progression.

## 2. Materials and Methods

### 2.1. Patient Inclusion Criteria and Sample Collection

A multicenter, prospective observational cohort study was conducted between September 2020 and July 2021 in Granada, Spain. Participants diagnosed with SARS-CoV-2 infection were enrolled from two hospitals—Hospital Universitario San Cecilio and Hospital Universitario Virgen de las Nieves—as well as from two primary healthcare centers, Salvador Caballero and Las Gabias. The study was conducted in accordance with the Declaration of Helsinki, and the protocol was approved by the Clinical Research Ethics Committee of Granada (CEIC; Omicovid-19 1133-N-20) on 18 June 2020. All patients provided written informed consent prior to participation. Recruited individuals had confirmed SARS-CoV-2 infection via RT-qPCR testing on nasopharyngeal swabs collected by trained medical staff. Participants did not present with other viral infections that could act as confounding variables. Based on clinical criteria outlined in established COVID-19 treatment guidelines [18], patients were stratified into two severity categories: mild (n = 24) and severe (n = 31). The mild group included symptomatic individuals (e.g., fever, cough, headache, gastrointestinal symptoms, and anosmia) without dyspnea, abnormal imaging, or signs of respiratory compromise. The severe group included those with significant respiratory distress (≥30 breaths/min), oxygen saturation ≤93% at rest, PaO_2_/FiO_2_ ≤ 300 mmHg, or patients requiring ICU support due to respiratory failure, shock, or multi-organ dysfunction.

Stool samples from the severe cohort were collected by healthcare staff, whereas patients with mild symptoms provided self-collected stool samples at home due to pandemic-related restrictions. Stool samples were collected in collection tubes containing preservative media (OMNIgene•GUT, DNAGENOTEK, Ottawa, ON, Canada) and stored. On the other hand, blood samples were drawn into BD Vacutainer SSTII Advance tubes (Becton Dickinson, Franklin Lakes, NJ, USA). Samples were centrifuged for 10 min at 2450 rpm, and the supernatant was collected. Both fecal and blood samples were stored at −80 °C until processing and analysis.

### 2.2. Plasma Metabolomic Sample Processing

Proteins from serum samples were precipitated by adding acetonitrile (AcN) at a ratio of 1:8 (sample:AcN), followed by vortexing for 2 min. The mixture was then centrifuged at 15,200 rpm for 10 min at 4 °C. The resulting supernatant was transferred into HPLC-grade vials and evaporated to dryness using a GeneVac HT-8 evaporator (Savant, Holbrook, NY, USA). Dried extracts were reconstituted in a 50:50 (*v*/*v*) solution of AcN and water containing 0.1% formic acid, and vortexed for 1 min prior to analysis. Then, metabolite profiling was carried out using a high-resolution liquid chromatography–mass spectrometry system (LC-HRMS), consisting of an Agilent 1290 LC system coupled to a TripleTOF 5600 mass spectrometer (AB SCIEX, Concord, ON, Canada) equipped with an electrospray ionization (ESI) source operating in positive ion mode. The mobile phases used were eluent A (0.1% formic acid in water/acetonitrile, 90:10 *v*/*v*) and eluent B (0.1% formic acid in acetonitrile/water, 90:10 *v*/*v*). The gradient program was as follows: 1% B from 0.00 to 0.50 min; increased to 99% B from 0.50 to 11.00 min; held at 99% B until 15.50 min; returned to 1% B at 15.60 min; and maintained at 1% B until 20.00 min. The flow rate was set at 300 μL/min.

Additionally, mass calibration was automatically performed every 10 injections to ensure accuracy. Organic solvent (OS) blanks and quality control (QC) samples were injected throughout the run sequence. QC samples were prepared by pooling equal volumes of all serum specimens included in the study and were analyzed every 10 injections to monitor system performance and signal stability. OS blanks were used alongside QCs to identify any background contamination from solvents or the extraction process and to assess potential carryover effects.

### 2.3. Metabolomics Bioinformatic Analysis

Raw LC-HRMS data were processed using PeakView (version 1.0 with Formula Finder plugin version 1.0, AB SCIEX, Concord, ON, USA) and MarkerView software (version 1.2.1, AB SCIEX, Concord, ON, USA) to assess retention time (RT), mass-to-charge ratio (*m*/*z*), and extract ion intensities. Feature extraction was conducted over a retention time window of 1–18 min, with a minimum signal intensity threshold of 200 counts per second (cps). To distinguish true biological signals from background noise and potential contaminants, an additional filtering step was performed by comparing the signals from organic solvent (OS) controls with those from the experimental groups.

MetaboAnalystR (v.3.0) was used for functional pathway prediction applying the Mummichog algorithm (*p*-value cutoff = 1 × 10^−5^, MEN analysis). Metabolites were annotated by exact *m*/*z* and RT match, keeping the lowest mass error, and mapped to KEGG pathways. An additional filter step based on fold change (>1.5) and *p*-value (<0.05) was applied only for downstream visualization and selection of representative putative metabolites. For visualization, PCA was performed on Pareto-scaled intensities, and selected metabolites were represented as boxplots, annotated with their respective pathways to support biological interpretation.

### 2.4. Microbial 16S Sample Pre-Processing, Library Preparation, and Next-Generation Sequencing

Microbial DNA from stool samples was collected following the protocol previously described by Rodríguez et al. [19]. Then, library preparation was performed as follows: DNA was quantified using the Qubit dsDNA HS Assay Kit (12640ES60, Yeason Biotechnology, Shanghai, China), and total DNA was amplified by targeting the V3–V4 hypervariable regions of the bacterial 16S rRNA gene. Quality control of the amplified products was carried out using a high-throughput Invitrogen 96-well E-Gel system (Thermo Fisher Scientific, Waltham, MA, USA). PCR products were pooled into a single plate and normalized using the Invitrogen SequalPrep 96-well Plate Kit. The pooled libraries were then sequenced on an Illumina MiSeq platform.

### 2.5. Microbiota Bioinformatic Analysis

Analysis was performed following the protocol previously described by Garcia-Garcia Jorge et al. [20]. Briefly, quality assessment and adapter and removal of low-quality bases were performed using *FastQC* and *Trimmomatic*, respectively [21,22]. The resulting filtered reads were processed in *QIIME2* (Northern Arizona University, Flagstaff, AZ, USA), and amplicon sequence variant (ASV) generation was performed using the *DADA2* plugin [23]. Finally, taxonomic classification was assigned using the SILVA reference database (version 138, 99% full-length sequences) [24]. 

Alpha and beta diversity indices, as well as microbial composition, were calculated using the *Phyloseq* package in R [25]. Beta diversity differences were assessed using permutational multivariate analysis of variance (PERMANOVA), as implemented in the *vegan* package. Multivariable linear models were implemented using MaAsLin2 to assess associations between microbial taxa and clinical variables while adjusting for potential confounders. Differentially abundant taxa were identified via linear discriminant analysis effect size (LEfSe), using an LDA score threshold of 4, facilitated by the *microbiome* package.

### 2.6. Statistical Analysis

All analyses and visualizations were performed using R software. The distribution and homogeneity of variances for numerical clinical variables were evaluated using the Shapiro and Levene Test, respectively (*stats* and *car* packages). In accordance, variables with normal distribution were presented as mean ± standard deviation (SD), while non-normally distributed variables were reported as median and interquartile range (IQR). Group comparisons were conducted using a t-test for parametric data or a Wilcoxon test for non-parametric data. Alternatively, categorical variables were presented as proportions, and group differences were assessed using Fisher’s exact test.

Multivariable analysis was conducted for microbiota and plasma metabolites using linear models, including disease severity, age, sex, gastrointestinal disorders, diabetes, obesity, cardiomyopathy, and antibiotic treatment as covariates. This allowed for the assessment of the effect of COVID-19 severity while accounting for potential cofounding by demographic and treatment-related variables.

For correlation analyses, Spearman’s correlation coefficients were calculated. Correlation matrices were visualized with the corrplot package. Finally, a *p*-value < 0.05 was considered statistically significant.

All scripts, processed data, and model outputs are available upon request.

## 3. Results

### 3.1. Study Cohort Description

For this study, a total of 55 patients were enrolled and divided into two groups based on COVID-19 severity. The mild group consisted of 24 patients, while the severe group comprised 31. The severe group was characterized by more pronounced symptomatology and a demographic profile summarized in an older and male population (Table 1). These findings are consistent with previous studies that have established the role of age and gender on COVID-19 severity [26]. Furthermore, patients with a severe condition presented a significant difference in the presence of cardiomyopathy, which has already been related to a poor prognosis of COVID-19 [27]. In terms of clinical laboratory parameters, patients with severe disease exhibited significantly elevated levels of C-reactive protein (CRP), D-dimer, ferritin, and platelets, which have been widely recognized indicators of disease severity in COVID-19 [28,29,30].

Taken together, the distribution of patients in our cohort adequately reflects the clinical stratification based on COVID-19 severity, supporting the validity of subsequent analyses. As a result, subsequent analysis of microbial and metabolomic data was adjusted for key covariates, including age, sex, comorbidities, and antibiotic treatment, in order to accurately identify associations with SARS-CoV-2 severity.

### 3.2. SARS-CoV-2 Infection Modifies Gut Microbiota Composition, Promoting an Impact on Symptomatology

**Figure 1:**
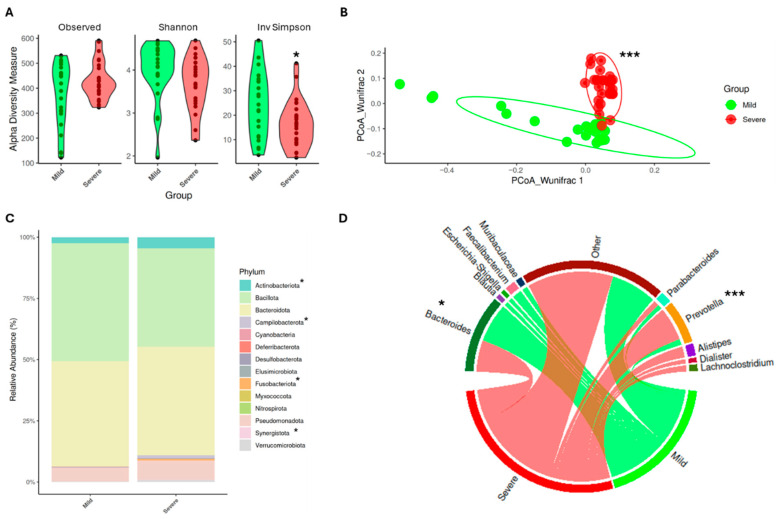
COVID-19 severity implies changes in gut microbiota composition. (**A**) Alfa diversity index analysis. (**B**) Principal Component Analysis (PCoA) for weighted unifrac distance. (**C**) Relative abundance at phylum level. (**D**) Relative abundance at genus level. Values are displayed as mean ± SD. Differences are represented by: * *p* < 0.05; *** *p* < 0.001. PERMANOVA test was employed to determine PCoA differences and multivariable regression models for alfa diversity and microbial relative abundance differences.

As previously reported, COVID-19 severity has been associated with alterations in gut microbiota composition [31]. Our findings are in line with these observations as disease severity was accompanied by changes in alpha and beta diversity of gut microbiota (Figure 1A). Concretely, patients with worse conditions exhibited a significant reduction in the inverse Simpson index (*p* < 0.05), indicating lower microbial diversity compared to patients with mild symptoms. In addition, beta diversity analysis (Figure 1B) revealed significant differences in microbial community composition between the two groups (*p* < 0.001), further supporting the impact of disease severity on the microbiome.

The changes in microbiota composition were further evaluated at the phylum and genus levels. Notably, patients with severe disease presented a significant increase in the relative abundance of *Actinobacteriota*, *Campilobacterota*, *Fusobacteriota*, and *Synergistota* (*p* < 0.05) (Figure 1C). Once again, these results agree with previous studies, where higher levels of *Actinobacteriota* and *Campilobacterota* have been associated with poor clinical outcomes [32]. Moreover, in nasopharyngeal microbiota, *Fusobacterium nucleatum* has been identified as an opportunistic pathogen in COVID-19 patients. Thus, its increased presence in the gut microbiome of severe cases may be linked to exacerbation of disease symptoms [33].

At the genus level (Figure 1D), only those genera with a relative abundance greater than 5% and present in at least 80% of patients were considered. Those genera that did not meet these criteria were categorized as “Other”. Based on this selection, patients with mild symptoms showed a significantly higher relative abundance of *Bacteroides*, *Blautia*, *Escherichia-Shigella*, *Faecalibacterium*, and *Muribaculaceae* (*p* < 0.05). In contrast, patients with severe COVID-19 exhibited increased levels of *Prevotella* (*p* < 0.001), *Alistipes*, *Dialister*, and *Lachnoclostridium*. Among the genera enriched in the mild group, except for *Escherichia-Shigella*, all of them have been associated with a protective role against infections. For instance, some species of *Bacteroides* are increased when SARS-CoV-2 viral load is reduced, suggesting a potential protective role [34]. Both *Faecalibacterium* and *Blautia* have been extensively described for their role in mitigating COVID-19 severity, particularly *F. prausnitzii*, which produces short-chain fatty acids (SCFAs) and may promote the secretion of anti-inflammatory cytokines [35]. Finally, although limited information is available regarding *Muribaculaceae*, some in vivo studies have proved their role in preventing COVID-19 severity [36].

On the other hand, *Alistipes* and *Dialister* are recognized as commensal bacteria. However, *A. finegoldii* has been positively correlated with inflammatory biomarkers that are also linked to increased COVID-19 severity [37]. Nevertheless, *Lachnoclostridium* is considered an opportunistic pathogen with a potential pro-inflammatory role that has also been found in the microbiota of SARS-CoV-2 infected patients in a previous work carried out by our research group [20]. Notably, *Prevotella* was significantly enriched in patients with severe COVID-19. Although it is a common genus in the human microbiota, several studies have linked *Prevotella* to pro-inflammatory responses. For instance, *Prevotella* species can activate toll-like receptor 2 (TLR2), leading to the increased production of IL-1β and other inflammatory cytokines, which may contribute to disease severity in viral infections [38]. Furthermore, in COVID-19 patients, increased abundance of *Prevotella* proteins have been correlated with promotion of viral infection [39]. These findings suggest that the expansion of *Prevotella* in severe cases may play a role in the exacerbation of inflammatory responses, potentially worsening disease prognosis.

Regarding genera classified as “Other”, a markedly distinct microbial profile was observed between patients with mild and severe symptomatology (Supplementary Figure S1). The mild group showed an increase in *Alloprevotella*, *Coprococcus*, *Dorea*, and *Roseburia*. All of them have been associated with a better prognosis as they are known producers of short-chain fatty acids (SCFAs), which exert immunomodulatory effects [40,41,42]. Meanwhile, the severe group presented an increase in *Anaerococcus*, *Corynebacterium*, *Enterococcus*, *Finegoldia*, and *Staphylococcus*. These genera have previously been linked to adverse clinical outcomes in COVID-19 patients. For instance, *E. faecium* has been found to be enriched in severe cases and is associated with lymphopenia, a known marker of poor prognosis [43,44].

Detailed relative abundances and statistical significance (*p*-values) for each genus can be found in the Supplementary Material.

**Figure 2:**
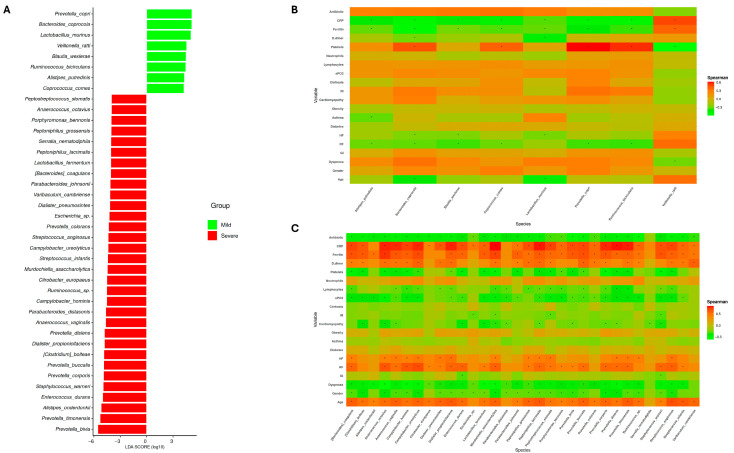
Specific bacteria related to COVID-19 severity correlate with the clinical status of patients. (**A**) LEfSe plot of taxonomic biomarkers from gut microbiota of SARS-CoV-2 infected patients (*p* value = 0.01 and linear discriminant analysis (LDA) value = 4). (**B**) Correlation plot of identified biomarkers from mild patients and clinical variables. (**C**) Correlation plot of severe biomarkers and clinical variables. Significant results from Spearman correlation test (*p* value < 0.05 and correlation value ≤−0.25 or ≥0.4) are represented with black asterisks.

Next, identification of specific biomarkers from each condition was done through a linear discriminant analysis (LEfSe). Consequently, while only 8 species were associated with mild conditions, 26 species were identified as potential predictors of COVID-19 severity (Figure 2A). Interestingly, mild species such as *Alistipes putredinis*, *Blautia wexlerae*, and *Coprococcus comes*, as well as *Ruminococcus* sp. abundance, have been correlated with a more favorable prognosis of SARS-CoV-2 infection [44,45,46] (Figure 2B). In addition, potential biomarkers identified in severe patients may be considered valid, given that several have been previously reported in relation to COVID-19 progression (Figure 2C). This is the case for *Prevotella* species (*P. colorans*, *P. disiens*, *P. buccalis*, *P. corporis*, *P. timonensis*, and *P. bivia*) as mentioned above [39]. For example, *Dialister pneumosintes* has been reported as an etiological agent of severe pneumonia, suggesting its potential role in aggravating COVID-19 severity [47]. Similarly, *Campylobacter ureolyticus*, an opportunistic pathogen, has been isolated from patients with severe COVID-19 manifestations [32].

The results obtained are consistent with previous studies, suggesting that during SARS-CoV-2 infection, patients who develop severe disease experience alterations in gut microbiota characterized by the emergence of pro-inflammatory bacteria and opportunistic pathogens. The higher numbers of species detected in severe patients are biologically plausible, as a more severe condition is likely to induce greater alterations in microbial composition, reflecting that disease severity itself influences bacterial community structure. Importantly, LEfSe analysis identifies features based on both statistical significance and effect size, helping to mitigate potential bias due to class imbalance. Therefore, the species identified through this model could be implemented as predictors of COVID-19 severity at the time of infection.

### 3.3. Severity Associated with SARS-CoV-2 Infection Modifies Blood Metabolome Profile

Gut microbiota composition is not the only factor that has been investigated in relation to COVID-19 pathology. Blood metabolite profile has also been examined, as circulating metabolites can exert significant effects on human health [48]. Consequently, several groups have already explored alterations in blood metabolite profiles associated with COVID-19. For example, Liu J. et al. revealed that severity induced an increase in AMDP, dGMP, and sn-glycero-3-phosphocoline in the blood of patients with severe COVID-19 compared to those with mild symptoms [49].

**Figure 3:**
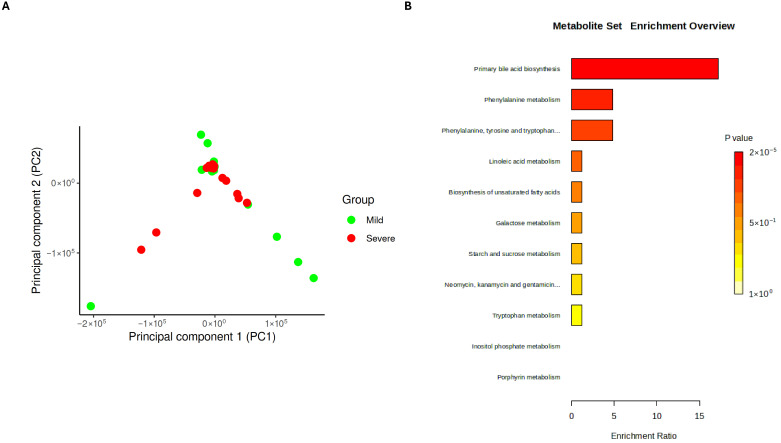
Blood metabolome profile of mild and severe COVID-19 patients. (**A**) Principal Component Analysis (PCA) plot showing the distribution of blood metabolite profiles between mild and severe patients. (**B**) Functional enrichment plot displaying the main metabolite sets differentially, based on untargeted LC-HRMS data.

Subsequently, in the present study, the plasma metabolite profile was assessed using liquid chromatography to determine whether disease severity is associated with metabolic alterations. Principal component analysis (PCA) revealed a general clustering trend among the samples. Nevertheless, some samples from patients with mild symptoms were clearly distinct from those of patients with severe disease, suggesting that specific metabolite changes may be linked with disease severity (Figure 3A). Notably, independently of severity, SARS-CoV-2 infection was associated with an increase in metabolites related to primary bile acid and unsaturated fatty acids biosynthesis as well as phenylalanine, linoleic acid, galactose, starch, sucrose, tryptophan, inositol phosphate, and porphyrin metabolism (Figure 3B). These pathways have already been explored in the COVID-19 context. For instance, elevated levels of bile acids have been reported in infected individuals [12], along with an increase in unsaturated fatty acids [14]. Additionally, disturbances in tryptophan metabolism have been associated with inflammation and immune dysregulation in COVID-19 patients [50].

**Figure 4:**
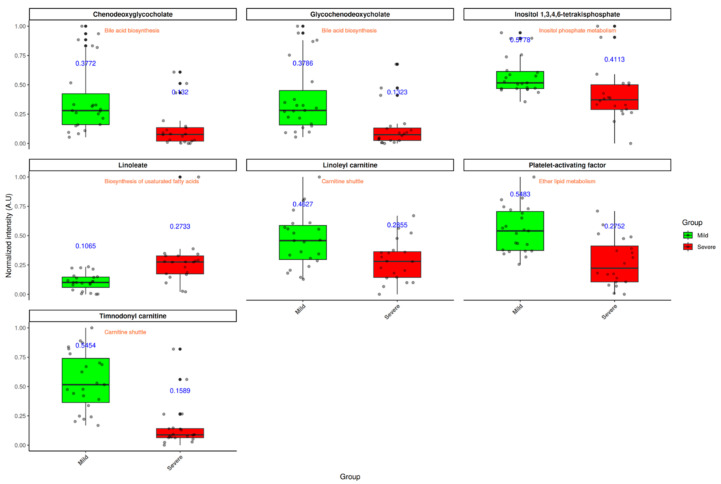
COVID-19 severity induces changes in blood metabolic profile. Boxplots represent normalized intensity of significantly altered metabolites. Multivariable regression models were carried out to find statistical differences. *p*-values were adjusted using Benjamini–Hochberg method. Mean values are displayed in blue while metabolite-associated pathway is annotated within each panel.

Based on the identification of the most upregulated metabolic pathways, the influence of disease severity on individual metabolites within these pathways was subsequently evaluated. These compounds represent putative metabolite annotations based on accurate mass and retention time. Thus, their assignment and downstream biological interpretation should be regarded as tentative. Taking into account statistically significant differences among clinical groups, only seven possible metabolites were identified as differentially abundant: chenodeoxyglycocholate, glycochenodeoxycholate, inositol 1,3,4,6-tetrakisphosphate (InsP4), linoleate, linoleyl carnitine, platelet-activating factor (PAF), and timnodonyl carnitine. Among these, only linoleate was observed to be increased in patients with more severe symptoms (Figure 4). A complete list of all the identified metabolites is provided in the Supplementary Material.

These findings should be interpreted with caution, as the reported levels of certain metabolites in the literature remain uncertain. For instance, with respect to bile acids—specifically chenodeoxyglycocholate and glycochenodeoxycholate—Valdés et al. reported an increase in their levels correlating with COVID-19 severity [15]. In contrast, in the present study, these bile acids were found to be increased in patients with mild symptoms. This discrepancy may reflect the fact that certain bile acids have been investigated for their antiviral properties, including their ability to inhibit viral replication [51], which could contribute to a more controlled disease progression. In addition, methodological differences between studies, including platform, ionization mode, and sample preparation, could have also contributed to these differences in observed bile acid levels. Regarding carnitine derivatives, the literature also presents conflicting evidence, with some studies reporting elevated levels and others indicating a decrease in the context of SARS-CoV-2 infection [52,53]. In alignment with the findings of Valdés et al., our results demonstrate a significant increase in acylcarnitines among patients with mild disease [15]. This suggests that the potential protective effect of carnitines in COVID-19 might be mediated through ketone bodies derived from carnitine metabolism, as these molecules are known to exert both immunomodulatory and anti-inflammatory effects [54].

On one hand, InsP4 is a well-recognized second messenger derived from inositol (vitamin B8), involved in diverse signaling pathways, including the regulation of intracellular calcium flux and immune cell function [55]. Given that inositol has demonstrated antiviral activity against multiple viruses [56], the increased levels of InsP4 observed in patients with mild symptoms may contribute to modulating immune responses, potentially exerting a protective effect through immunoregulatory mechanisms. On the other hand, PAF is a potent lipid mediator produced by different cell types (endothelial cells, neutrophils, platelets, etc.), which is characterized by its proinflammatory and thrombotic properties [57]. In the present study, patients of mild symptomatology showed a significant increase in PAF levels. Although this finding may appear contradictory—given that elevated PAF levels have been previously associated with increased morbidity and mortality in COVID-19 [58]—it is conceivable that this early rise reflects the initial activation of T and B lymphocytes during the immune response. In contrast, the reduced PAF levels observed in patients with severe disease may be attributed to the administration of glucocorticoid therapy or to enhanced catabolic activity of enzymes involved in PAF degradation, as previously proposed by Antonopoulou et al. [59].

Finally, patients with severe COVID-19 exhibited a significant increase in linoleate levels, which is consistent with previously reported findings. In the context of SARS-CoV-2 infection, unsaturated fatty acids have been identified as biomarkers associated with disease severity. Specifically, linoleic epoxides, which are metabolized into diols, promote inflammation [60]. In fact, several studies have tried to improve the condition of patients through omega-3 administration in order to reduce linoleate-derived molecules [61].

In summary, the metabolomic profile observed in this study is consistent with previously reported findings, supporting the potential utility of these specific circulating metabolites as reliable biomarkers for assessing COVID-19 severity.

### 3.4. Potential Bacterial Indicators of COVID-19 Severity Display Significant Associations with Metabolomic Profiling

**Figure 5:**
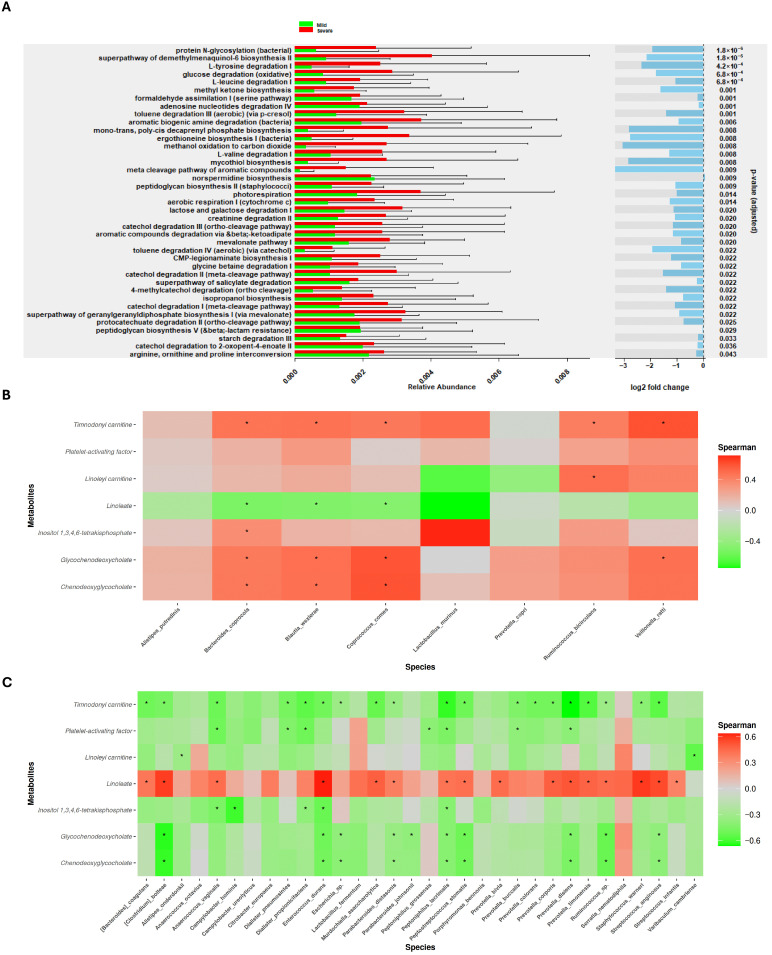
COVID-19 severity induces an increase in predicted bacterial metabolomic pathways alongside a strong positive correlation with linoleate, a metabolite related to inflammation. (**A**) Top metabolic pathways predicted with PICRUSt2. (**B**) Correlation plot of increased bacteria and plasma metabolites in mild patient group. (**C**) Correlation plot of increased bacteria and plasma metabolites in severe patient group. Significant results for PICRUSt2 were inferred from a LINDA analysis. In the case of correlation, Spearman test (*p* value < 0.05 and correlation value≤ −0.6 or ≥0.6) is represented with black asterisks.

In the present study, rather than directly measuring microbiota-derived metabolites, we predicted the principal bacterial metabolic pathways associated with the previously identified microbial species using the PICRUSt2 software (v 2.5.2) (Figure 5A). It should be noted that PICRUSt2 outcome represents predicted metabolic capabilities rather than directly measured microbial metabolites or metagenomic functions. Consequently, these results indicate potential relationships rather than confirmed microbial activities. Therefore, functional predictions revealed a set of metabolic pathways significantly enriched in patients with severe COVID-19. Among these, amino acid degradation pathways—such as leucine catabolism and the interconversion of arginine, ornithine, and proline—were presumed to be notably overrepresented, potentially reflecting the increased metabolic demands imposed by infection. Indeed, microbial infections have been associated with amino acid depletion and the loss of host body proteins [62]. Additionally, the enrichment of pathways related to oxidative glucose metabolism could suggest a metabolic shift in bacterial energy production that may affect host physiology. Experimental studies using *Caenorhabditis elegans* have shown that glucose processing by commensal *E. coli* can impair host health-span and lifespan by enhancing oxidative stress and the accumulation of advanced glycation end-products [63]. Finally, the predicted enrichment of microbial pathways involved in the degradation of aromatic xenobiotic compounds—such as toluene and catechol—is of particular interest. The catabolism of these compounds has been linked to pro-inflammatory responses [64], which could exacerbate the systemic inflammation observed in severe COVID-19 cases. Collectively, these predicted functional alterations in the gut microbiome may contribute to disease severity through mechanisms involving increased catabolic stress, redox imbalance, and enhanced inflammatory signaling.

Additionally, pathways associated with peptidoglycan biosynthesis and bacterial protein *N*-glycosylation were probably found to be more active in patients with severe disease. Many glycoproteins have been identified in pathogenic bacteria such as *Pseudomonas aeruginosa*, *Haemophilus influenzae*, *Campylobacter jejuni*, and *Clostridium* spp. [65]. Therefore, the observed increase in *Campylobacter* and *Clostridium* species in severe COVID-19 cases may promote bacterial glycosylation activity, potentially influencing host–pathogen interactions and modulating immune responses. Furthermore, the enrichment of pathways involved in the biosynthesis of vitamins—menaquinone (vitamin K2) and ergothioneine—suggests an attempt to mitigate oxidative stress, as both compounds are known for their antioxidant properties [66]. In parallel, the upregulation of CMP-legionaminate, geranylgeranyl diphosphate biosynthesis, and the mevalonate pathways points toward enhanced bacterial surface remodeling and isoprenoid production, a characteristic of pathogenic bacteria [67]. These findings, based on predicted microbial functions from PICRUSt2, underscore the potential metabolic plasticity of the gut microbiota in the context of severe inflammatory conditions, highlighting its potential role in shaping host immune responses and systemic physiological processes.

Beyond predicting the molecular pathways potentially involved, it was also essential to explore the associations between gut microbiota composition and plasma metabolites. On one hand, Figure 5B displays the correlations between bacterial species enriched in patients with mild symptoms and the identified metabolites. Notably, except for *Alistipes putredinis*, *Lactobacillus murinus*, and *Prevotella copri*, the remaining species exhibited positive correlations with metabolites known to have pro-resolving or anti-inflammatory properties, as previously discussed. Conversely, *Bacteroides coprocola*, *Blautia wexlerae*, and *Coprococcus comes* showed negative correlations with linoleate—a metabolite found to be more abundant in severe cases—suggesting a potential depletion of beneficial microbial taxa during the progression to more severe disease.

On the other hand, Figure 5C shows the correlations between microbial species enriched in severe COVID-19 patients and the measured plasma metabolites. Most of these bacteria demonstrated negative correlations with key metabolites typically associated with metabolic homeostasis and anti-inflammatory activity. Of particular interest, linoleate—the only metabolite significantly elevated in severe patients—exhibited positive correlations with multiple taxa linked to severe disease, reflecting a possible potential role in COVID-19 pathophysiology and its association with disease severity. These results highlight linoleate as an interesting candidate whose functional impact and mechanistic contributions should be further studied to confirm or refute the observed results.

## 4. Conclusions

Taken together, the findings presented in this study suggest that the alterations in gut microbiota composition observed in COVID-19 patients with severe symptoms may influence the circulating metabolomic profile. These microbiota-associated changes may modulate host inflammatory responses and immune regulation through shifts in bacterial metabolism and metabolite production. Consequently, the bacterial taxa and metabolites identified herein could be considered as candidate markers for assessing disease severity in patients infected with SARS-CoV-2.

## Figures and Tables

**Table 1:** Clinical data of recruited patients. Data is represented as median and SD or median and interquartile range (IQR), depending on normality distribution. Depending on this value, *t*-test or Wilcoxon test was employed to determine statistical differences. Categorical variables are displayed with %, and Fisher’s test was used for assessing statistical differences. * = *p* < 0.05; ** = *p* < 0.01 and *** = *p* < 0.001.

	Mild (n = 24)	Severe (n = 31)	
**Clinical variables**			***p* value**
Median Age (IQR), years	45 [35; 54]	65 [57; 71] ***	0.001
Male (%)	33	71 ***	0.007
**Symptomathology (%)**			
Gastroinstestinal alteration (Yes)	21	32	
Dyspnoea (Yes)	25	84 ***	0.001
Respiratory rate (High)	17	61 ***	0.001
sPO_2_ (Low)	25	71 ***	0.001
Heart rate (High)	17	55 ***	0.008
**Comorbidities (%)**			
Asthma (Yes)	13	6	
Cardiomyopathy (Yes)	13	42 *	0.02
Cirrhosis (Yes)	8	0	
Diabetes (Yes)	17	26	
Renal injury (Yes)	13	0	
Obesity (Yes)	21	32	
**Plasma biomarkers**			
Median C reactive protein (IQR), mg/L	3.4 [2.8; 4]	162 [65.2; 210.4] ***	0.001
Median D-dimer (IQR), mg/L	0.4 [0.2; 0.9]	1.62 [0.92; 4.35] ***	0.001
Median ferritin (IQR), ng/L	157.2 [126.4; 179.7]	829.8 [488.6; 1376.9] ***	0.001
Median lymphocytes (IQR), 10^3^ µL	1.2 [0.6; 1.7]	0.6 [0.4; 0.9]	
Median neutrophils (IQR), 10^3^ µL	6.04 [5.5; 6.6]	7.9 [5.4; 10.9]	
Median platelets (IQR), 10^3^ µL	272 [203; 342.5]	332 [322; 336] **	0.01
**Treatment (%)**			
Antibiotics (Yes)	0	58 ***	0.001

## Data Availability

The datasets supporting the findings of this study are openly available at FigShare: https://doi.org/10.6084/m9.figshare.30642548 (accessed on 18 November 2025).

## Supplementary Materials

The following supporting information can be downloaded at: https://www.mdpi.com/article/10.3390/medsci14010097/s1, Figure S1: Relative abundance of the genera named as “Other”; Supplementary Table S1: Complete list of metabolites detected in the study.
