# Supplementary material for: Microbiome–Metabolome Crosstalk as a Driver of COVID-19 Severity
Source: Med Sci (Basel). 2026 Feb 17;14(1):97. doi: 10.3390/medsci14010097 (PMC12921900; doi:10.3390/medsci14010097)
Supplement: Supplementary file 1 [file medsci-14-00097-s001.zip › medsci-4114636-supplementary/Supplementary material.pdf]

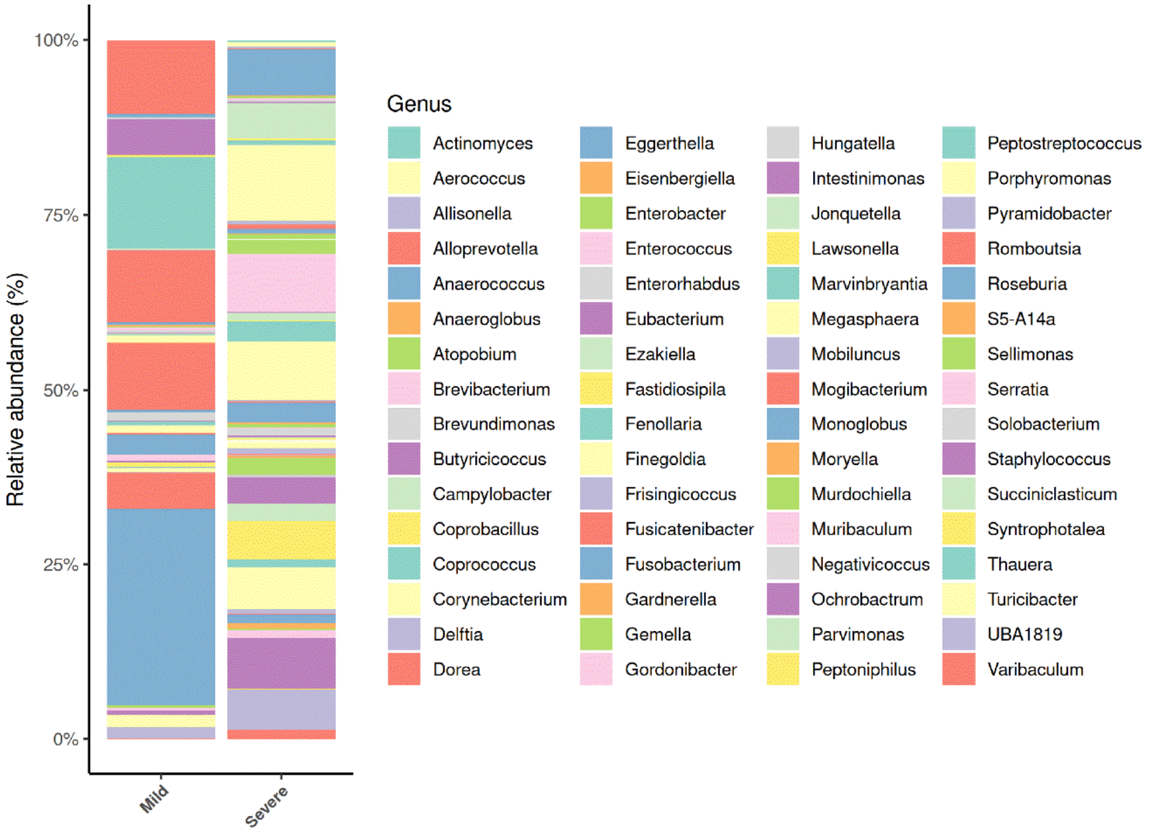

Supplementary Figure S1. Relative abundance of bacterial groups classified as “other” at the genus level, showing genera grouped under this category because their relative abundance was below 5% in 90% of the samples.

Supplementary Table S1. Complete list of metabolites detected in the study, based on the identification of the most upregulated metabolic pathways and subsequent evaluation of disease severity–associated differences among clinical groups.

Identified metabolites	Molecular pathway
L-Phenylalanine	Phenylalanine metabolism
5-Methoxyindoleacetate	Tryptophan metabolism
D-Glucose	Glycolysis / Gluconeogenesis
L-Tryptophan	Tryptophan metabolism
Linoleic acid	Biosynthesis of unsatturated fatty acids
Docosahexaenoic acid	Biosynthesis of unsatturated fatty acids
Chenodeoxyglycocholate	Biosynthesis of unsatturated fatty acids
Linoleyl carnitine	Carnitine shuttle
Elaidic carnitine	Carnitine shuttle
Glycochenodeoxycholic acid	Primary bile acid biosynthesis
Timnodonyl carnitine	Biosynthesis of unsatturated fatty acids
Inositol 1,3,4,6-tetrakisphosphate	Inositol phosphate metabolism
Cervonyl carnitine	Carnitine shuttle
Docosa-4,7,10,13,16-pentaenoyl carnitine	Biosynthesis of unsatturated fatty acids
1-acylglycerophosphocholine	Glycerophospholipid metabolism
Tetracosapentaenoyl carnitine	Carnitine shuttle
Platelet-activating factor	Ether lipid metabolism
Biliverdin	Porphyrin metabolism
